# Unusual Presentation of Diffuse Large B-Cell Lymphoma With Splenic Infarcts

**DOI:** 10.1177/2324709617690748

**Published:** 2017-02-01

**Authors:** Vivek Kumar, Parita Soni, Vishangi Dave, Jonathan Harris

**Affiliations:** 1 Maimonides Medical Center, Brooklyn, NY, USA

**Keywords:** diffuse large B-cell lymphoma, splenic infarct, lymphadenopathy, mass lesion, biopsy, diagnosis, presentation

## Abstract

A 67-year-old man presented with a 3-day history of abdominal pain, fever, and significant weight loss over 2 months. Physical examination revealed left upper quadrant tenderness, hepatomegaly, splenomegaly, and bilateral pitting edema but peripheral lymphadenopathy was absent. Laboratory tests showed anemia, thrombocytopenia, elevated prothrombin time (PT), partial thromboplastin time (PTT), and increased lactate dehydrogenase (LDH). PTT was corrected completely in mixing study. Further workup for the cause of coagulopathy revealed decreased levels of all clotting factors except factor VIII and increase fibrinogen levels, which ruled out disseminated intravascular coagulation (DIC). Flow cytometry of peripheral blood was normal. Contrast-enhanced computed tomography (CECT) revealed splenomegaly with multiple splenic infarcts without any mediastinal or intraabdominal lymphadenopathy. Further investigations for infective endocarditis (blood cultures and transthoracic echocardiography) and autoimmune disorders (ANA, dsDNA, RA factors) were negative. The patient received treatment for sepsis empirically without any significant clinical improvement. The diagnosis remained unclear despite extensive workup and liver biopsy was conducted due to high suspicion of granulomatous diseases. However, the liver biopsy revealed high-grade diffuse large B-cell lymphoma (DLBCL). Unfortunately, patient died shortly after the diagnosis. Here we report a case of high-grade DLBCL with hepatosplenomegaly and splenic infarcts in the absence of any lymphadenopathy or focal lesions. This case highlights the fact that unusually lymphoma can present in the absence of lymphadenopathy or mass lesion mimicking autoimmune and granulomatous disorders. The diagnosis in these cases can only be made on histology, and hence the threshold for biopsy should be low in patients with unclear presentations and multiorgan involvement.

## Introduction

Splenic infarcts are rare and result from the occlusion of splenic artery or one of its branches with an embolus secondary to septic emboli from the vegetations on the mitral or aortic valves or less frequently by localized thrombosis as seen in leukemia, myeloproliferative disorders, sickle cell disease, or in vasculitis.^1,2^ It is described only rarely with diffuse large B-cell lymphoma (DLBCL).^3^ We report a rare case of splenic infarcts in a patient with DLBCL. Besides splenic infarcts, this case also illustrated a rare presentation of DLBCL, characterized by the absence of peripheral and internal lymphadenopathy, focal lesions, and the presence of diffuse hepatic involvement.

## Case Presentation

**Figure 1.**
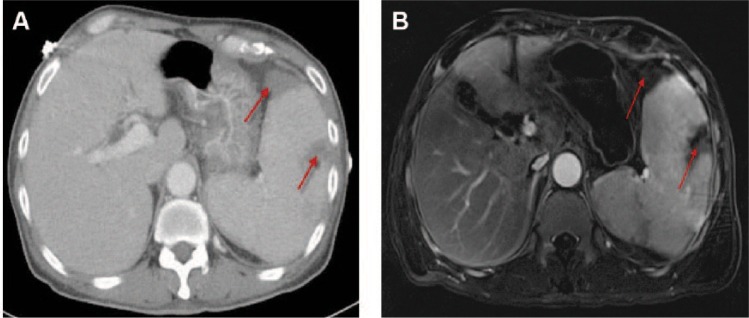
(A) Axial view of the computed tomography image of the abdomen showing splenomegaly with multiple splenic infarcts (shown by the arrows). (B) Magnetic resonance imaging of the abdomen showing splenomegaly with multiple splenic infarcts (shown by the arrows).

A 67-year-old male of Asian descent with a history of hypertension, presented to the emergency room with sharp severe abdominal pain and fever for 3 days. He also complained of worsening shortness of breath, dry cough, swelling over bilateral lower extremities, and significant weight loss for last ~6 weeks. On physical examination, crepitations over bilateral chest, left upper quadrant tenderness, hepatomegaly, splenomegaly and bilateral 3+ pitting edema were present without any skin lesions or peripheral lymphadenopathy. Blood tests showed anemia (hemoglobin 10.3 mg/dL), thrombocytopenia (platelets 80 000/µL), marked hypoalbuminemia (albumin 1.6 g/dL), increased lactic acid (6 mmol/L), erythrocyte sedimentation rate (ESR) (60 mm/h), lactate dehydrogenase (LDH) (471 IU/L) and ferritin (829 ng/mL). The coagulation profile was considerably deranged with prolonged prothrombin time (PT) and partial thromboplastin time (PTT). Further evaluation of coagulopathy revealed diminished levels of all clotting factors except factor VIII and increased fibrinogen levels, which ruled out disseminated intravascular coagulation (DIC). Liver enzymes, kidney function, hepatitis panel, thyroid function, angiotensin converting enzyme levels (ACE), lipase, human immunodeficiency virus (HIV), antinuclear antibody (ANA), antidouble stranded DNA antibodies (dsDNA), antiphospholipid antibodies (APLA), anti-cardiolipin antibodies and rheumatoid (RA) factor were within the normal range. Flow cytometry of peripheral blood smear did not exhibit leukemia or any other lymphoproliferative disorder. Contrast-enhanced computed tomography (CECT) of the chest, abdomen, and pelvis revealed splenomegaly with multiple splenic infarcts in the absence of any mediastinal or intraabdominal lymphadenopathy (Figure1A). These findings were also confirmed on magnetic resonance imaging (MRI) of the abdomen (Figure 1B). A 7-mm hypodensity was reported in the posterior left hepatic lobe, which could not be characterized due to its very small size. Hepatic and portal venous Doppler examination was negative for any thrombus. Workup for infective endocarditis (IE) including 3 sets of blood cultures and transthoracic echocardiogram (TTE) was also negative. Patient received treatment for sepsis empirically with broad spectrum antibiotics but without any significant clinical improvement. The differential diagnosis at this stage were the following:

1. *IE*: The clinical presentation of IE is variable. The insidious onset of illness with duration lasting for 6 weeks, nonspecific symptoms and weight loss strongly suggested IE. Hepatomegaly with painful splenomegaly is also a classical finding in IE, though it is not present in all the cases. The lab abnormalities like normocytic normochromic anemia and raised ESR also supported IE. Nevertheless, in this patient IE was ruled out due to negative blood cultures and normal TTE. Due to high index of suspicion, echocardiogram was repeated after 1 week but it remained negative.
2. *Autoimmune disorders:* The nonspecific symptoms of several weeks duration, involvement of multiple organs and weight loss were suggestive of an underlying autoimmune granulomatous disorder like sarcoidosis. However, absence of autoimmune markers and histopathological findings on liver biopsy ruled out autoimmune etiology.
3. *Malignancy:* Advanced age with nonspecific symptoms and weight loss with subacute course may be seen in solid organ tumors (kidneys, colon, lung, and liver) or hematological malignancies like leukemia and lymphoma. Nonetheless, absence of mass lesion on imaging, normal flow cytometry on peripheral blood and absence of any lymphadenopathy made likelihood of malignancy very low initially. At the outset, most of the workup was directed to rule out IE and autoimmune diseases. The DLBCL could only be diagnosed after the liver biopsy.

**Figure 2.**
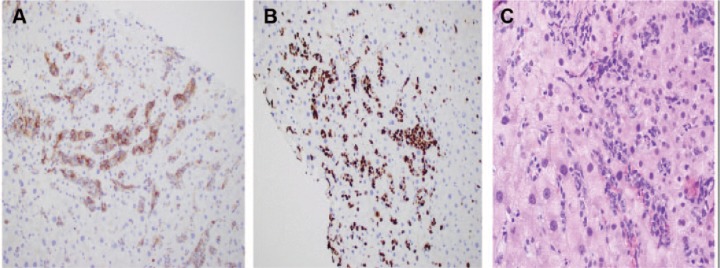
(A) Liver biopsy showing hepatic parenchyma with predominantly sinusoidal prominence of lymphoid cells with modest periportal infiltrates. (B) Immunohistochemistry (IHC) with hematoxylin counterstain showing diffuse CD20 (B-cell) positivity of tumor cells (20×). (C) Ki-67 immunostaining of lymphoma cells with a high proliferative rate (20×).

The diagnosis remained unclear despite extensive workup; thus, liver biopsy was planned to rule out granulomatous diseases. The patient underwent liver biopsy through the transjugular approach. The histopathology revealed normal hepatic parenchyma with predominantly sinusoidal prominence of lymphoid cells with modest periportal infiltrates (Figure 2A). On Immunohistochemistry (IHC), the lymphoid cells in sinusoidal and interstitial sites were diffusely positive for CD20 with a Ki-67 index of approximately 80% to 90 (Figure 2B and C) and were negative for CD3, CD4, CD5, CD56 and CD138. This established the diagnosis of DLBCL. The underlying liver parenchyma was normal without any evidence of hemophagocytosis. Shortly after the liver biopsy, the clinical condition of the patient worsened and he got intubated for respiratory distress and acute pulmonary edema (day 9 of hospitalization). Immediately after intubation, he suffered cardiac arrest and died. The autopsy could not be conducted due to the lack of consent from family.

## Discussion

The presentation of intermediate- and high-grade DLBCL is highly variable. Majority of patients present with peripheral and internal lymphadenopathy, and extranodal involvement is seen in up to one-third of these patients.^4^ Primary extranodal involvement with B symptoms such as fever, night sweats, and significant weight loss are frequently seen in patients with advanced or end-stage disease but they are rare on initial presentation.^4,5^ Splenic infarcts are also rare in DLBCL, and their presence often directs the workup towards IE or autoimmune disorders, which frequently delays the diagnosis of an underlying malignancy.^6^ The diagnosis of DLBCL in such unusual cases can only be established after the biopsy, which is often conducted to rule out other diseases.^7^ The prognosis of these patients depends on the stage, grade, and performance status but in general remains poor.^7^ Retrospectively this patient had B-symptoms (weight loss, nonspecific symptoms) for several weeks. The presence of hypoalbuminemia with coagulopathy in this patient suggested liver failure in the beginning. However, bilirubin and aminotransferase remained normal throughout the hospital stay. The hypoalbuminemia and coagulopathy most likely resulted from malnutrition with increased protein catabolism mediated by interferons and tumor growth factors.^8,9^ Severe hypoalbuminemia led to anasarca and pulmonary congestion. In fact, patient remained fluid overloaded despite rigorous diuresis and developed respiratory failure due to acute pulmonary edema as a terminal event.

Some rare forms of DLBCL, such as primary hepatic lymphoma and Asian variant of intravascular lymphoma (AIVL), can also present without lymphadenopathy and mass lesions. Primary hepatic lymphoma is an extranodal form of non-Hodgkin’s lymphoma (NHL) with primary site of origin in the liver.^4^ It is extremely rare and less than 100 cases have been described in the literature so far. It mostly affects middle-aged males and manifests clinically as abdominal pain, weight loss, splenic infarcts, and fever without any significant peripheral or internal lymphadenopathy.^4,6^ On imaging, a single large liver mass or multiple nodules mimicking metastatic disease are common, but less commonly it can also present with diffuse hepatic involvement. Serum LDH levels are often high with normal α-fetoprotein (AFP) and carcinoembryonic antigen (CEA).^10^ The diagnosis is established on finding lymphoma cells in the parenchyma, sinusoids and periportal regions on the liver biopsy. Its prognosis is dismal with average survival of approximately 6 months despite multi-agent chemotherapy. In our case, histopathology of liver was not suggestive of primary hepatic lymphoma. AIVL is another form that is marked by the presence of lymphoma cells exclusively in the lumen of small blood vessels, particularly capillaries.^11^ It is also an extranodal disease, mainly reported from southeast Asian region, which manifests without any mass lesions. The clinical presentation of typical intravascular lymphoma is highly variable and includes skin lesions, stroke, focal neurological deficits, and splenic infarctions.^11^ However, in AIVL, skin or neurological involvement is absent. In these patients bone marrow involvement and hemophagocytosis syndrome is seen more often.^11^ The diagnosis is established by skin or liver biopsy showing lymphoma cells localized to capillaries or sinusoids without portal or parenchymal involvement.^12^ The prognosis is poor regardless of combination chemotherapy. In this patient of Asian descent, absence of lymphadenopathy and presence of splenic infarcts mimicked this form, but on liver biopsy unlike in AIVL, lymphoma cells infiltrated the liver parenchyma.

## Conclusion

Splenic infarcts are uncommon and are usually manifestations of a broader spectrum of diseases. The absence of lymphadenopathy and mass lesions do not completely preclude the diagnosis of lymphoma. The unusual presentation of DLBCL usually simulates autoimmune and connective tissue disorders. The diagnosis in such cases can only be made on histology and therefore threshold for biopsy should be low in such cases.
